# Isolation and Characterization of Mannanase-Producing Bacteria for Potential Synbiotic Application in Shrimp Farming

**DOI:** 10.3390/ani12192583

**Published:** 2022-09-27

**Authors:** Witida Sathitkowitchai, Ponsit Sathapondecha, Pacharaporn Angthong, Yanee Srimarut, Yuwares Malila, Wuttichai Nakkongkam, Sage Chaiyapechara, Nitsara Karoonuthaisiri, Suttipun Keawsompong, Wanilada Rungrassamee

**Affiliations:** 1 National Center for Genetic Engineering and Biotechnology, 113 Thailand Science Park, Phahonyothin Road, Khlong Nueng, Khlong Luang, Pathum Thani 12120, Thailand; 2 Center for Genomics and Bioinformatics Research, Division of Biological Science, Faculty of Science, Prince of Songkla University, Hat Yai, Songkhla 90112, Thailand; 3 International Joint Research Center on Food Security, 113 Thailand Science Park, Phahonyothin Road, Khlong Nueng, Khlong Luang, Pathum Thani 12120, Thailand; 4 Institute for Global Food Security, Queen’s University Belfast, Biological Sciences Building, 19 Chlorine Gardens, Belfast BT9 5DL, UK; 5 Department of Biotechnology, Faculty of Agro-Industry, Kasetsart University, Chatuchak, Bangkok 10900, Thailand

**Keywords:** probiotics, mannanase, mannan-oligosaccharide, copra meal, aquaculture, penaeid shrimp, *Bacillus*, *Niallia*, *Penaeus monodon*

## Abstract

**Simple Summary:**

Mannooligosaccharides (MOS) can promote growth and immunity in aquatic animals. Although most commercial MOS are derived from yeast, the use of MOS from copra meal, a waste product of the coconut industry, is of interest. MOS from copra meal, when given as a dietary supplement to shrimp, could help improve resistance to pathogenic *Vibrio* and could also act as an immunostimulant. Our objective was to identify bacterial isolates that could be used as probiotics for shrimp and together with MOS as synbiotics to synergistically improve shrimp performance and feed utilization. In this study, two bacterial candidates, Man26 and Man122, were isolated from shrimp intestines and screened for mannanase, the enzyme for mannan digestion. The crude enzymes were evaluated for their biological properties and potential application in the digestion of feedstuffs *in vitro*. Both strains were able to produce and secrete mannanase to digest MOS and other mannan-rich materials and could tolerate a wide pH range. The addition of crude enzymes significantly increased the reducing sugars of copra meal, palm kernel cake, and soybean meal (*p* < 0.05) as well as protein release. The synergistic effect of our bacteria and MOS on shrimp growth performance will be further explored in the field.

**Abstract:**

Prebiotics such as mannan-oligosaccharides (MOS) are a promising approach to improve performance and disease resistance in shrimp. To improve prebiotic utilization, we investigated the potential probiotics and their feasibility of synbiotic use in vitro. Two bacterial isolates, Man26 and Man122, were isolated from shrimp intestines and screened for mannanase, the enzyme for mannan digestion. The crude mannanase from both isolates showed optimal activities at pH 8 with optimum temperatures at 60 °C and 50 °C, respectively. The enzymes remained stable at pH 8–10 for 3 h (>70% relative activity). The thermostability range of Man26 was 20–40 °C for 20 min (>50%), while that of Man122 was 20–60 °C for 30 min (>50%). The *V_max_* of Man122 against locust bean gum substrate was 41.15 ± 12.33 U·mg^−1^, six times higher than that of Man26. The *K_m_* of Man26 and Man122 were 18.92 ± 4.36 mg·mL^−1^ and 34.53 ± 14.46 mg·mL^−1^, respectively. With the addition of crude enzymes, reducing sugars of copra meal, palm kernel cake, and soybean meal were significantly increased (*p* < 0.05), as well as protein release. The results suggest that Man26 and Man122 could potentially be used in animal feeds and synbiotically with copra meal to improve absorption and utilization of feedstuffs.

## 1. Introduction

The global demand for seafood has increased significantly in recent years due to the rapid growth of the world’s population. Seafood production has become an important global market potential, with 85.3 million tons in 2019 and a growth of 3.7 percent compared to 2018 [1]. FAO estimates that seafood consumption will reach 180,504 thousand tons in 2030 [2]. The primary source of seafood still depends heavily on nature and marine fisheries, which limits the seafood production [3]. Therefore, it is challenging to meet the rapidly increasing global seafood demand to ensure stability, accessibility, and availability for food security.

Crustaceans are among the top three aquaculture production, accounting for 12.3% of the production in 2019 with 10.5 million tons [1]. Among the various crustacean species, shrimp production reached a new high of more than 336,000 tons in 2017 and 2018 [3]. However, shrimp production is not yet sustainable mainly due to the frequent occurrence of diseases such as white spot disease (WSD), acute hepatopancreatic necrosis disease (AHPND), yellowhead disease (YHD), and white feces disease (WFD) [4,5,6,7]. The disease outbreak affects mortality, growth rate, and deformations. Therefore, improving disease tolerance in shrimp production would be crucial to achieve sufficient seafood production for global demand.

One of the promising approaches to improve the growth performance and disease resistance of shrimp is functional feed additives [8,9]. Feed additives include immunostimulants, organic acidic essential oils, enzymes, probiotics, and prebiotics [10,11] which are commonly used to promote absorption, immune level, nutrient digestibility, and gut microbial balance [12]. In particular, gut microbiota has been associated with host health such as host performance and disease resistance in shrimp [12,13,14]. The gut microbiota has also been reported to influence host metabolism and body composition, vitamin synthesis, and prevent pathogen colonization [13,15,16]. Therefore, modulation of the gut microbiota by feed additives has attracted considerable interest. Prebiotics, complex soluble carbohydrates, have been shown to promote healthy microbial diversity in animal guts [17]. Inulin, β-glucan, fructo-oligosaccharides (FOS), gluco-oligosaccharides (GOS), and manno-oligosaccharides (MOS) have been widely used as prebiotics in aquaculture to improve survival, growth performance, and nutrient digestibility and to promote beneficial bacteria such as *Lactobacillus* and *Bifidobacterium* [18,19,20,21]. Among the different types of prebiotics, MOS can be obtained from yeast cell walls (*Saccharomyces cerevisiae*) or copra meal (by-product of the oil extraction from coconut) [22]. MOS have been shown to promote probiotics, modulate immune responses, and inhibit pathogen colonization in animals [23]. MOS from yeast cell walls (*S. cerevisiae*) have been reported to improve aquatic animal health, such as growth performance, gut health, and immune system [20,24,25,26,27]. MOS from copra meal have been also shown to improve survival rate and immune system in shrimp [28]. For example, green tiger shrimp (*Penaeus semisulcatus*) fed with MOS have improved growth performance, feed conversion, and survival rate [29]. In *Litopenaeus vannamei*, the effect of MOS as a prebiotic was reported to increase the number of beneficial bacteria, while reducing the incidence of potential opportunistic pathogens, such as *Vibrio*, *Shewanella*, and *Aeromonas* [27]. In addition to prebiotics, probiotics are the alternative to boost immunity and improve survival and gut microbial balance [30,31,32]. Many different genera of lactic acid bacteria (LAB), yeast, *Streptococcus*, *Enterobacter*, *Shewanella*, *Bacillus*, and *Lactobacillus* have been applied as probiotics in fish and shellfish [33,34,35].

Recently, much interest has been shown in using prebiotics and probiotics together as synbiotics to achieve synergistic effects on growth performance and disease resistance in shrimp [36]. Synbiotic has stronger positive effects on animal health than the use of prebiotics or probiotics alone [35]. In aquaculture, prebiotics and probiotics can be developed separately but applied synbiotically. To improve the sustainability and efficacy of the synbiotic approach, we were interested in selecting endogenous intestinal bacteria enriched in shrimp fed with previously known prebiotics, i.e., MOS from copra meal [28]. In this work, we screened bacterial isolates from shrimp intestine fed with MOS for a 6-week period. The bacterial candidates were selected based on their ability to produce mannanase, prebiotic digestive enzymes capable of digesting and utilizing MOS. The crude enzymes of each bacterial isolate were further characterized for their stability, efficiency, and potential application for breaking down various feed ingredients.

## 2. Materials and Methods

### 2.1. Experimental Animals and Diets

#### 2.1.1. Diets Supplemented with Mannan-Oligosaccharides (MOS)

The mannan-oligosaccharides (MOS) used in this study were obtained from the enzymatic digestion of copra meal as described in our previous study [28]. Four experimental diets were made by top-coating of commercial feed pellets (38% protein) with different concentrations of MOS: 0% (control diet), 0.2%, 0.3%, and 0.4% MOS.

#### 2.1.2. Shrimp Feeding Trial

Black tiger shrimp *P. monodon* post larvae were obtained from the local commercial farm in Pathum Thani (Thailand) and tested to be free from the following pathogens: WSSV, YHV, *Vibrio parahaemolyticus* AHPND, and *Enterocytozoon hepatopenaei*. The shrimp were reared at the Aquaculture Research and Service Development Team facility (National Center for Genetic Engineering and Biotechnology, Thailand) until reaching approximately 3 g in size. Six hundred shrimps were randomly distributed into 24 fiberglass tanks containing 390 L of 15 ppt-water water (25 shrimps/tank, 32 shrimp/m^2^), and 6 tanks were assigned to each of the four experimental diets (Figure S1). After 6 weeks of the feeding trial, 3 tanks of each experimental diet were designated for growth performance analysis, while the remaining tanks were further used in a pathogen challenge experiment.

Shrimp were fed five times a day to apparent satiation with daily adjustment. Water quality parameters were monitored and maintained: pH between 7.5–8.0, dissolved oxygen (DO) greater than 5 mg/L, temperature between 28–32 °C, total ammonia-nitrogen below 0.03 mg/L, nitrite-nitrogen below 1 mg/L, and alkalinity between 100–150 mg CaCO_3_/L.

All uses of animals in this experiment were approved by the National Center of Genetic Engineering and Biotechnology IACUC (Project code BT-Animal 24/2561)

### 2.2. Shrimp Survival Assessment under Vibrio harveyi Exposure

Growth performance, average daily growth, and feed conversion ratio were determined from 3 replicate tanks at the end of the feeding trial. To conduct the pathogen challenge experiment, *Vibrio harveyi* was cultivated according to the previously published method of Sritunyalucksana et al. [37]. After the 6-week feeding period, shrimp were challenged with pathogenic *V. harveyi* VH0 using an injection technique. For each dietary treatment, a total of 42 shrimps (14 shrimp from each replicate tank) were divided into two groups: challenged and control groups. Shrimp in the challenged group (7 shrimps/tank; 3 replicate tanks) were injected intramuscularly with a culture suspension of *V. harveyi* (approximately 1.4 × 10^6^ CFU/shrimp). The control group was injected with sterile saline water. Cumulative mortality was monitored for 96 h. During the challenge, shrimps remained fed with their corresponding experimental diet.

### 2.3. Tissue Sample Collection and Bacterial Screening

Intestine samples were collected from the group of shrimps fed with MOS and used as sources for isolation of mannanase-producing bacteria. Each shrimp intestine was chopped into fine pieces and added to 200 μL of 1X phosphate buffer pH 7.4. Each sample was homogenized using sterilized micro pestles. Homogenate sample (60 μL) was inoculated into 3 mL of M9 minimal media (3 mM Na_2_HPO_4_, 2 mM KH_2_PO_4_ 1 mM NaCl, 1 mM NH_4_Cl, 2 mM MgSO_4_, 0.1 mM CaCl_2_) with 1% MOS (copra meal hydrolysate) as a sole carbon source. The bacteria were grown under aerobic conditions by shaking at 250 rpm for 16–18 h at 30 °C.

For primary screening, M9 culture broth was serially diluted and spread on M9 agar containing 1% MOS and incubated at 30 °C for 24 h. The colonies were further screened on TCBS agar (Oxoid, UK). Our bacterial candidates were those that were able to grow on M9 containing 1% MOS as the sole carbon source but not on TCBS to avoid potential pathogenic *Vibrio*. The selected colonies were grown in LB containing 1% locust bean gum (LBG, Sigma-Aldrich, St. louis, MO, Sigma) at 30 °C for 24 h. Colonies with a clear zone indicating mannanase activity were confirmed by the 0.5% Congo red staining method [38,39] and bacterial candidates were stored in 20% glycerol at −80 °C.

### 2.4. Enzyme Production and Culture Conditions

Production of mannonate from the bacterial isolates was carried out in M9 minimal media (3 mM Na_2_HPO_4_, 2 mM KH_2_PO_4_ 1 mM NaCl, 1 mM NH_4_Cl, 2 mM MgSO_4_, 0.1 mM CaCl_2_, and 1% LBG as a carbon source) at 37 °C for 24 h. Briefly, a bacterium was inoculated to 100 mL at 3% (*v*/*v*). After 24 h of growth at 37 °C and 220 rpm, the culture was centrifuged at 10,000 g, 4 °C for 15 min and the cell-free supernatant, namely our crude enzyme, was collected and stored at –20 °C.

### 2.5. Enzyme Activity Assay and Determination of Optimal Reaction Time, pH, and Temperature

Enzyme activity assay was performed according to the 3,5-dinitrosalicylic (DNS) method [40] using LBG as a substrate. Crude enzyme (100 μL) was incubated with 1% (*w*/*v*) of LBG in 100 mM glycine-NaOH (pH 8.0) at 60 °C for 10 min for Man26 and 50 °C for 20 min for Man122, which were optimal conditions for each enzyme identified in this study. Reducing sugars were then determined using 1% (*w*/*v*) DNS reagent and the absorbance of reaction mixture was measured at a wavelength of 540 nm. One unit (U) of the enzyme activity is defined as the amount of enzyme required to produce 1 μmol of mannose per minute under the specific conditions. Enzyme activity was calculated and expressed as the number of activity units per milliliter of crude enzyme (U/mL).

To determine optimum incubation time, the mannanase activity was measured at 5, 10, 20, 30, 40, 50, and 60 min in 100 mM sodium phosphate buffer (pH 7). As for pH, the enzyme activity was determined using different ranges of the pH buffer as follows: 100 mM citrate buffer (pH 3–6), 100 mM sodium phosphate buffer (pH 6–8), and 100 mM glycine-NaOH buffer (pH 8–11). The effect of temperature on enzyme activity was examined by incubating at different temperatures from 20 °C to 90 °C. All enzyme activity assay was carried out using the optimal conditions for Man26 and Man122, respectively. The highest activity was used as 100% baseline for a relative comparison of the other activities; therefore, the relative activities were calculated using the following equation:

The relative activity (%) = (Enzyme activity of each point/The highest enzyme activity) × 100

### 2.6. Determination of Stability and Kinetics

The pH stability was determined by pre-incubation of the enzymes at different pH values (pH 3–10) for 3 h at 30 °C compared to 0 h, and then the mannanase activity was determined and compared. Thermal stability was evaluated by pre-incubating the enzymes at various temperatures from 20 °C to 80 °C for 1 h before determining mannanase activity. The highest activity of each condition was used as 100% baseline activity for relative comparison.

Enzyme kinetics were determined according to Lineweaver–Burk kinetics under optimal conditions at LBG concentrations of 0, 1, 2, 4, 6, 8, 10, 12, 14, 16, 18, and 20 mg/mL. *K*_m_ and *V*_max_ values were calculated using the KaleidaGraph program version 3.5 (Reading, PA, USA).

### 2.7. Determination of Substrate Specificity and Effects of Different Chemicals

The substrate specificity of crude mannanase was determined by incubating the crude enzymes with 1% (*w*/*v*) of various substrates under our identified optimal conditions. The substrates include LBG (Sigma-Aldrich, St. Louis, MO, USA), guar gum, (Sigma-Aldrich, St. Louis, MO, USA), carboxymethyl cellulose (Sigma-Aldrich, St. Louis, MO, USA), xylan from larchwood (Sigma-Aldrich, St. Louis, MO, USA), xylan from oat spelts (Sigma-Aldrich, St. Louis, MO., USA), xylan from birchwood (Sigma-Aldrich, St. Louis, MO, USA), Avicel (Merck, Darmstadt, Germany), and potato starch (Sigma-Aldrich, St. Louis, MO, USA). Then, the reducing sugar was determined using DNS method as described above. The highest mannanase activity was used as a baseline for relative activity comparison.

The effect of different chemicals on enzyme activity was performed in the presence of the tested chemicals in the enzyme activity assays under optimal conditions for each crude extract. The enzyme activity without any additional chemical was used as a baseline for relative activity comparison.

### 2.8. Degradation of Feedstuffs

Degradation of feedstuffs by crude mannanase was examined in maize, broken rice, and soybean meal from a swine farm in Nakorn Prathom, palm kernel cake (PKC) from a palm oil mill in southern Thailand, copra meal (CPM) from a coconut oil factory in Thailand, and commercial shrimp feed (INTEQC, Thailand). The reaction of the enzymes consisted of 100 enzyme units at optimal temperature in glycine-NaOH pH 8 (the optimal pH) with 1% and 5% of different substrates (modified from Persons, 1984). The process was performed with each substrate (1% (*w*/*v*) and 5% (*w*/*v*)) in a total volume of 50 mL. In addition, the reaction mixtures were collected after 24 h and subsequently boiled at 100 °C for 15 min to stop the hydrolytic process, followed by filtration with filter paper grade 1 (Whatman, Germany). The filtrates were analyzed for reducing sugars (RS) and total sugars (TS) and measured by DNS method [40] and the phenol-sulfuric acid method [41], respectively. Protein release was measured using the Bradford protein assay [42].

### 2.9. Statistical Analysis

All data were analyzed using the statistical software package SPSS version 17. Statistical significance was determined using one-way analysis of variance (ANOVA) for enzyme characteristics. Differences between samples and controls for digestibility of feedstuffs were determined using a paired-sample *t*-test. All differences were considered statistically significant when *p* < 0.05.

## 3. Results

### 3.1. Growth Performance, Survival Rate of Shrimp under Normal Condition and under Exposure to Pathogenic Vibrio harveyi

**Figure 1:**
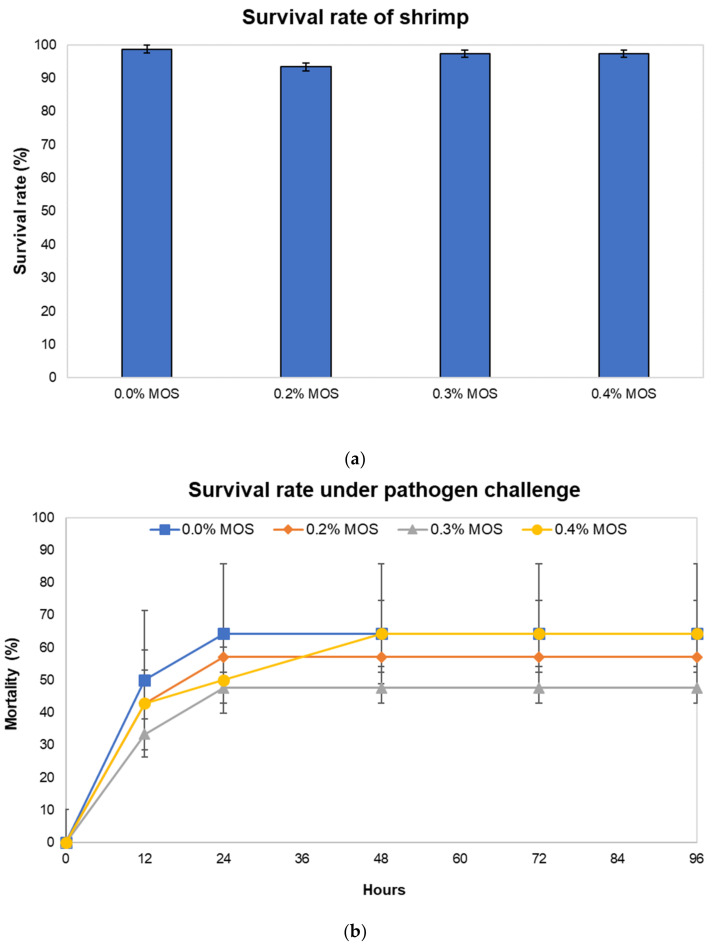
The effect of MOS on survival rate and the survival rate under the *Vibrio harveyi* challenge in shrimp fed with diets containing 0%, 0.2%, 0.3%, and 0.4% MOS. (**a**) The effect of MOS on survival without the pathogen challenge. (**b**) The effect of MOS on survival rate under the pathogen challenge. Error bars represent standard deviations calculated from triplicate samples (n = 3).

The 6-week feeding experiment was conducted with juvenile *Penaeus monodon* to investigate the effects of mannan-oligosaccharides (MOS) from copra meal as a feed additive on shrimp growth performance (Figure S2), in which our results showed no significant difference in the final weight, average daily growth, and feed conversion ratio. The survival rate during the feeding period and mortality rate under pathogen exposure were further determined (Figure 1). The survival rate of shrimp did not differ significantly among groups fed with diets containing 0%, 0.2%, 0.3%, and 0.4% MOS, indicating that MOS from copra meal had no harmful side effects on shrimp (Figure 1a). Although the groups supplemented with 0.2% and 0.3% MOS showed lower mortality rates under exposure to *V. harveyi* than the control group (Figure 1b), the mortality rates were not significantly different among the treatments (*p* > 0.05).

### 3.2. Screening of Bacterial Isolates with Potential Function to Hydrolyze MOS

The shrimp intestines were collected from the groups fed with an MOS-supplemented diet and used as sources for bacterial screening. A total of 440 bacterial colonies grown on M9 agar containing 1% MOS as a sole carbon source were obtained. The bacteria were further screened for their potential mannanase production by producing clear zones when growing on LB agar containing LBG, a rich source for mannan. Among a total of 122 colonies with clear zones on the LBG agar plate, the two isolates consistently showing mannanase activity were selected in this study, and they were renamed as Man26 and Man122, respectively (Figure S3). We previously determined complete full genome sequences of our bacterial candidates, Man26 and Man122, in which they showed similarity to *Niallia* sp. and *Bacillus* sp., respectively [43]. The morphological characteristics of the two isolates showed that both Man26 and Man122 were Gram positive with a rod shape (Table 1). The colony of Man26 had a white and shiny colony while Man122 were white with a dull texture. Both isolates exhibited catalase activity at 24 and 48 h (Table 1).

### 3.3. Enzymatic Characterization of Mannanase

**Figure 2:**
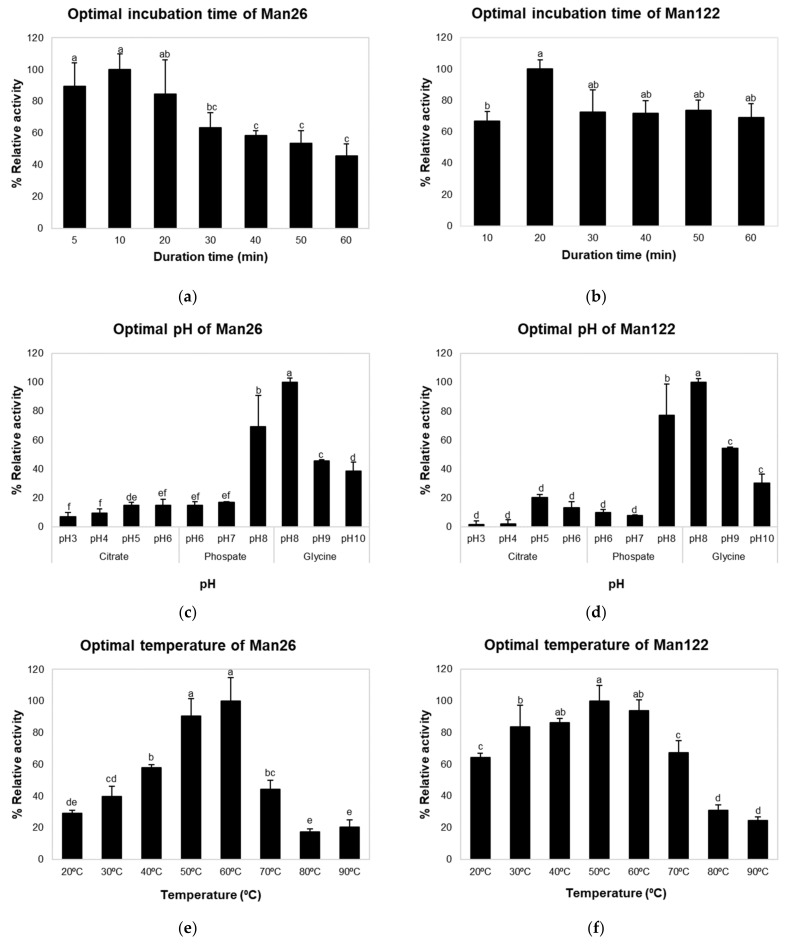
The optimal conditions for crude mannanase from Man26 and Man122. Optimal time of Man26 (**a**) and Man122 (**b**), optimal pH of Man26 (**c**) and Man122 (**d**), and optimal temperature of Man26 (**e**) and Man122 (**f**). Different superscripts represent significant differences (*p* < 0.05). Error bars represent standard deviations calculated from triplicate samples (n = 3).

The effects of time, pH, and temperature on crude mannanase activities of Man26 and Man122 were investigated (Figure 2). The optimal times for Man26 and Man122 were 10 and 20 min, respectively (Figure 2a). Mannanase activity at pH values ranging from 3.0 to 10.0 showed that the maximum activities of crude enzymes from Man26 and Man122 were significantly observed at pH 8.0 (*p* < 0.05) (Figure 2b). At pH 8.0, the activities of both crude enzymes in 0.1 M glycine-NaOH (100% relative activity) were higher than 0.1 M phosphate buffer (69.14% and 77.32%). The activities of both crude enzymes were higher under alkaline conditions than under acidic conditions. To determine the effect of temperature on enzyme activity, crude Man26 and Man122 were incubated in a temperature range of 20 °C to 90 °C (Figure 2c). Crude mannanase from Man26 and Man122 was significantly most active at 50 °C and 60 °C, respectively (*p* < 0.05). The crude mannanase activity of Man26 increased gradually from 20 °C to 50 °C but showed a decreasing trend when the temperature was above 60 °C. On the other hand, the crude mannanase activity of Man122 showed an increasing trend with an increasing temperature but declined after 60 °C (Figure 2c).

**Figure 3:**
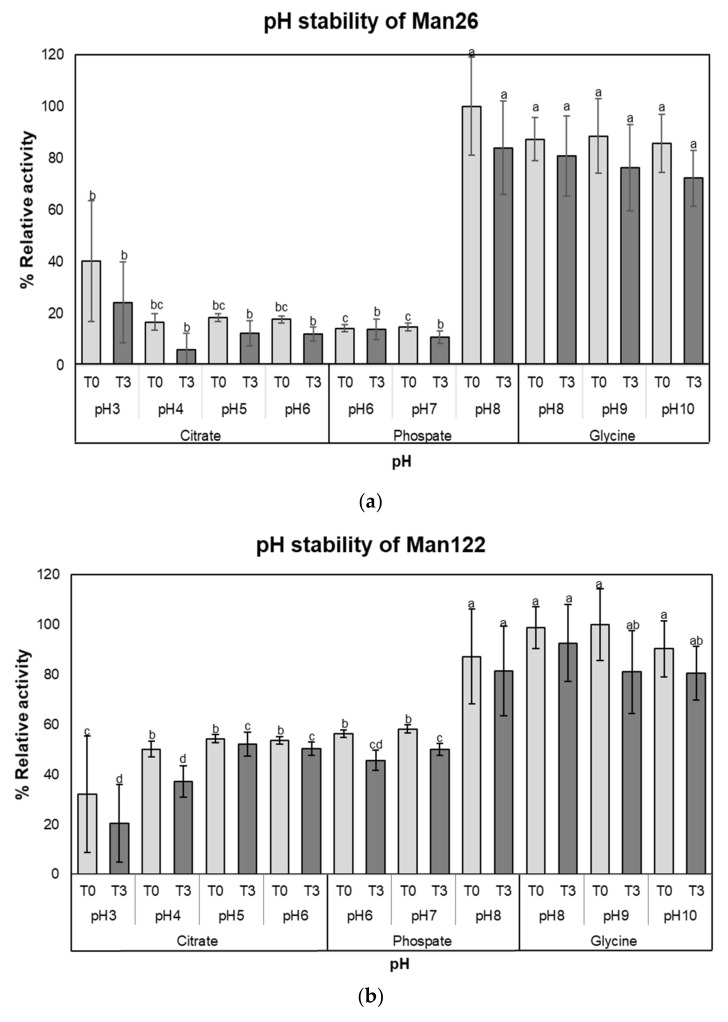

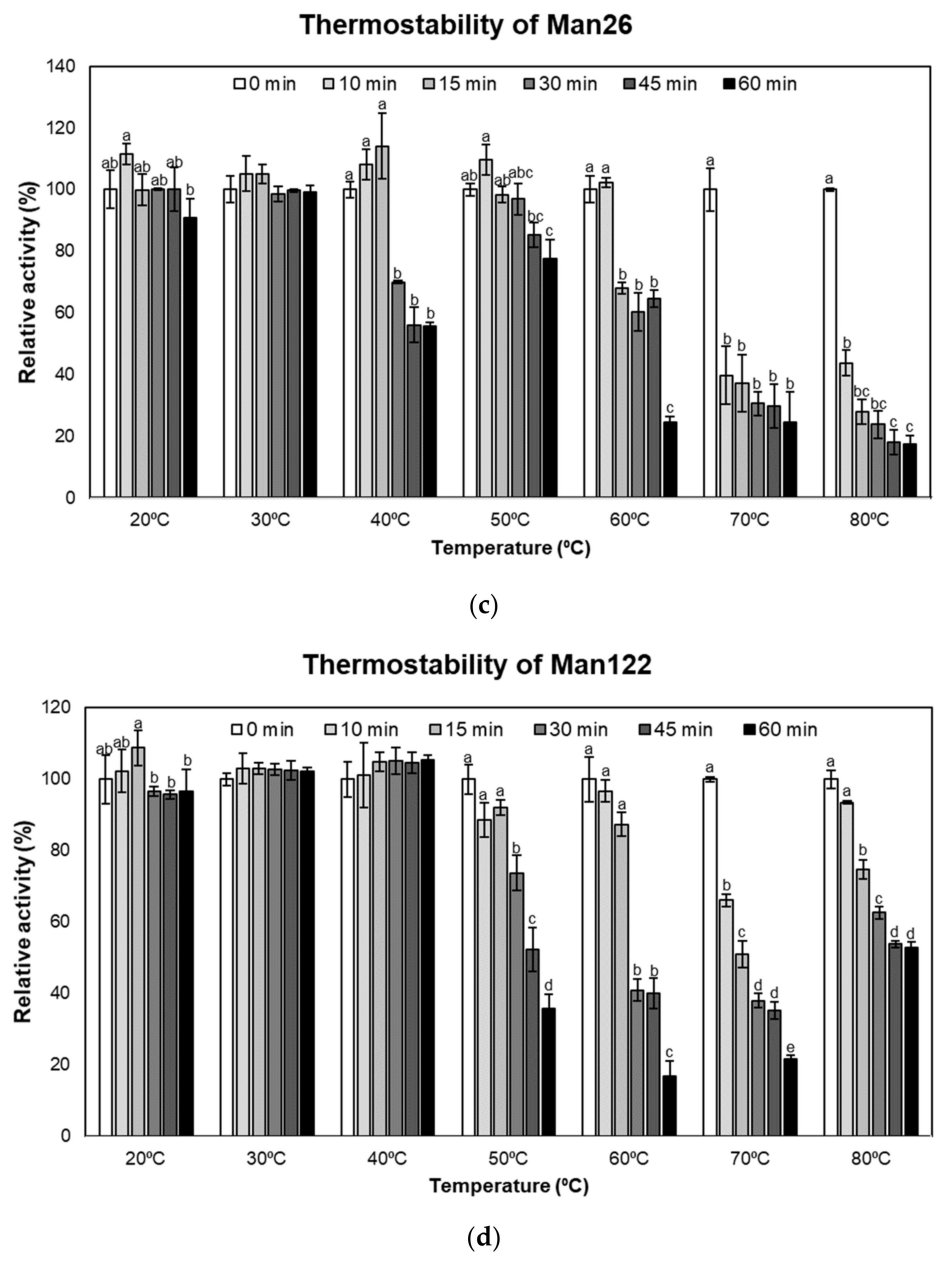
The stability of crude mannanase enzymes from Man26 and Man122. (**a**) pH stability of Man26, (**b**) pH stability of Man122, (**c**) thermostability of Man26, and (**d**) thermostability of Man122. Error bars represent standard deviations calculated using triplicate samples (n = 3). Different letters above each bar denote pH stability at each time point (T0 or T3), while thermostability is indicated within each temperature.

Both crude enzymes were stable over a wide pH range in an alkaline condition, with more than 80% activity at pH 8.0 to 10.0 (*p* < 0.05). Even at low pH values of pH 3.0, 4.0, and pH 5.0, crude mannanase from Man26 was 24.29 ± 15.58%, 6.14 ± 6.35%, and 12.35 ± 4.76% of relative activity, respectively (Figure 3a). On the other hand, crude mannanase from Man122 showed relative activity of 20.40 ± 7.79%, 37.23 ± 2.82%, and 52.26 ± 7.03% under pH values of 3.0, 4.0, and pH 5.0, respectively (Figure 3b). Nevertheless, the relative activity of both crude enzymes was significantly higher under alkaline conditions (pH 8 to pH 10) than under acidic and neutral conditions (pH 3 to 7) (Figure 3a and b).

To evaluate the thermostability of Man26 and Man122 (Figure 3c,d), the crude enzymes of both isolates were preincubated for 0 to 60 min in a different temperature range from 20 to 80 °C before determining their activities. Man26 and Man122 showed a similar trend in thermostability; the longer the enzymes were preincubated at 50 °C or higher, the lower the enzyme activities were observed. In Man26, we found that enzyme activity decreased significantly after 60 min incubation at 20 °C (*p* < 0.05) (Figure 3c). At 30 °C, all incubations were not significantly different, whereas the enzyme was stable for only 15 min incubation at 40 °C. As with Man122 (Figure 3d), enzyme activities were relatively stable at 20 to 40 °C. On the other hand, sharp decreases were observed when the enzyme was preincubated at 50 to 80 °C from 30 min (Figure 3d). Nevertheless, more than 20% of the activity was retained at all the experimental temperatures. The results indicate that the crude mannanases of Man26 and Man122 were still active after 1 h of preincubation at different temperatures in the range of 40–80 °C.

The *V_max_* of crude mannanase from Man122 against LBG substrate was 41.15 ± 12.33 U·mg^−1^, which was six times higher than that of crude mannanase of Man26 (Table 2). The *K*_m_ of the two crude enzymes were 18.92 ± 4.36 mg·mL^−1^ and 34.53 ± 14.46 mg·mL^−1^, respectively.

The crude mannanases from Man26 and Man122 showed the highest activity on LBG, followed by Guar gum for Man26 and potato starch for Man122 (*p* < 0.05) (Table 3). Our results showed that the crude enzymes were also able to partially hydrolyze cellulose substrates (CMC and Avicel) and other hemicellulose substrates from natural products such as xylans from larch wood, birch wood, and oat husks. Among the different substrates, the highest activity was observed in LBG, a mannan-rich substrate, indicating that the crude mannanase of both isolates was highly specific for β-mannan substrates (Table 3). When comparing the two isolates, the crude mannanase from Man26 showed significantly higher activity in guar gum and avicel, while the crude mannanase from Man122 showed significantly higher activity in CMC, larchwood, and oat spelts.

To determine the effects of different chemicals on mannanase activity, various buffers were added to the reaction, and their relative activity was investigated (Table 4). Compared with the control samples without chemicals, the crude mannanases of Man26 and Man122 were significantly inhibited by 1%, 5%, and 10% SDS (*p* < 0.05). The mannanase activity of Man26 was slightly increased by 10 mM MgCl_2_. In addition, 10 mM KCl, 10 mM MgSO_4_, and 10 mM NaCl had a small effect on Man26 enzyme activity with relative activities greater than 90% in the presence of these chemicals. Amounts of 10 mM CaCl_2_, 10 mM EDTA, 10 mM Na_2_SO_3_, and 10 mM NH_4_Cl had a moderate effect on activity of Man26. For Man122, all chemicals did not enhance activity, but 10 mM KCl and 10 mM NaCl had a slight inhibition of enzyme activity with relative activities greater than 85% in the presence of these chemicals, while 10 mM CaCl_2_, 10 mM MgCl_2_, 10 mM MgSO_4_, and 10 mM NH_4_Cl had a moderate effect on the enzyme activity (60–70%). In addition, the mannanase activity of Man122 was significantly reduced by MgCl_2_, MgSO4, Na_2_SO_3_, NH_4_Cl, EDTA, and SDS compared with that of Man26 (*p* < 0.05), suggesting that the mannanase of Man122 was more sensitive to the chemicals than that of Man26.

### 3.4. Enzyme Activity on Selected Carbohydrate Feedstuffs

To determine the ability of crude mannanases from Man26 and Man122 isolates to break down feedstuffs, palm kernel cake (PKC), copra meal (CPM), maize, broken rice, soybean meal, and commercial shrimp feed pellets were used (Table 5 and Table 6). The levels of reducing sugar and total sugar and protein releases during incubation with the Man26 crude enzyme are shown in Table 5. Considering PKC and CPM, the feedstuffs composed of mannan structures, crude mannanase of Man26 could breakdown the PKC and CPM at both 1% and 5%, resulting in significantly higher release content of reducing sugar and protein compared to their control counterparts (no enzyme) (*p* < 0.05). However, this crude mannanase could only increase the release of total sugar when the enzyme was incubated with 1% CPM. When the enzyme of Man26 was incubated with other feedstuffs, it significantly increased the release of protein in all feedstuffs compared with their control counterparts (*p* < 0.05). The release of reducing sugar was increased for maize, broken rice, and soybean (*p* < 0.05). When the percentage of substrate was increased from 1% to 5%, the amount of nutrient releases tended to increase in a dose-dependent manner (*p* < 0.05).

A similar trend was observed when the feedstuffs were incubated with crude mannanase from Man122 (Table 6), but with a slight difference. Significant increases in the release of reducing sugar, total sugar, and protein were observed when 1% and 5% of PKC and CPM were incubated with the crude enzyme from Man122 (*p* < 0.05). The release of total sugar was also significantly higher in most of the tested feedstuffs, except for 1% of soybean, when the crude mannanase was present in the reaction. However, only soybean (1% and 5%) with the addition of the enzyme showed significant increases in digestibility compared to their control counterparts (*p* < 0.05). The significant increase in reducing sugar, total sugar, and protein upon incubating with crude extracts from both Man26 and Man122 suggested that our isolates could break down complex feedstuffs and possibly can improve nutrient uptake by shrimp.

## 4. Discussion

Most synbiotic applications in aquaculture are often performed in animal studies without understanding the mode of action of the “probiotics of choice” and their prebiotics; hence, efficiency and synergistic effects may not be optimal and sustainable [44]. In this work, we determined the activity of our probiotic candidates and the digestion of prebiotics in vitro to understand their mechanisms prior to field application. Our bacterial candidates were isolated from the intestines of black tiger shrimp fed with MOS of copra meal (CPM). The bacteria were selected for their ability to utilize MOS as a sole carbon source, suggesting that they produced some enzymes for this process. The selected isolates would be promising probiotic candidates for synbiotic use with MOS as prebiotics to improve gut health and growth performance of shrimp. Here, we also validated the effect of MOS from copra meal on increasing pathogen resistance, which is consistent with previous reports showing that MOS can enhance the growth performance of shrimp such as weight gain, feed conversion ratio, growth rate, and disease resistance [9,27,45].

The source of the bacterial isolate is one of the important factors to be considered for sustainability of probiotics used in aquaculture. Moreover, one of the modes of action of probiotics is to produce digestive enzymes to increase feed utilization and digestibility [36,46,47,48,49]. In this study, our bacterial candidates were isolated from shrimp intestines, showing that they could thrive under shrimp environments. They were screened for their ability to produce carbohydrases for hydrolyzing prebiotics, particularly MOS, and to utilize feedstuffs. The feasibility of applying these bacterial strains will further be investigated in the animal trial. From previous studies, non-starch polysaccharides in cell walls are associated with water absorption and an increase in the viscosity of digested feedstuff, which affects utilization of proteins, starch, and other nutrients. Therefore, the addition of exogenous enzymes, such as carbohydrase, to animal feed could improve the interaction between feed–enzyme interaction and consequently increase feed utilization [50,51,52]. The carbohydrases commonly used in animal production are xylanase, glucanase, amylase, galactosidase, pectinase, cellulase, and mannanase [50,53]. Here, we were particularly interested in mannanase due to its ability to breakdown mannan to produce MOS as immunostimulants in aquaculture [54,55]. The crude enzymes of our bacterial isolates were able to digest MOS, and mannanase activities were relatively higher under alkaline conditions (pH 8–10). Alkali-stable enzymes are becoming increasingly important in the food and feed industry, particularly in prebiotic production [56,57]. Therefore, in addition to a direct application in shrimp culture, our alkali-stable mannanase could have a dual application as a biocatalyst in the production of MOS [58]. Various studies have shown that feeds, such as CPM and PKC, pre-treated with mannanase improve nutrients such as protein release and could decrease crude fiber, hemicellulose, and cellulose [59,60]. Our crude extracts of Man26 and Man122 were able to hydrolyze the carbohydrate structure of feedstuffs, especially CPM and PKC, which are mainly composed of >80% mannan, including galactomannan [61,62]. As expected, crude enzymes prepared from our bacterial isolates showed higher substrate specificity for CPM than for PKC because PKC was more complex. Nevertheless, our crude extracts were able to hydrolyze CPM and PKC more than other feedstuffs because CPM and PKC contained more hemicellulose than those in maize, broken rice, and soybean meal [61,62]. Therefore, Man26 and Man122 could potentially be used to improve feed utilization in shrimp.

Our bacterial isolates, Man26 and Man122, showed multifaceted activity such as cellulase, amylase, and xylanase. Man26 and Man122 were able to digest the carbohydrate structure feedstuffs and release the nutrients in vitro. Our results suggest that these bacterial isolates, which were endogenous in the intestine of shrimp, have the potential to improve host and gut health as probiotics. Moreover, the fact that these mannanases of Man26 and Man122 were able to hydrolyze various mannan polymers (LBG, CPM, PKC) makes them promising candidates to be used synbiotically to enhance feed digestion and nutrient absorption. Their synbiotic applications are under further investigation.

## 5. Conclusions

In the present study, bacterial strains were isolated from the intestines of *P. monodon* juveniles fed with a diet supplemented with MOS and characterized for their mannanase activity. Two candidates, Man26 and Man122, were obtained and identified as *Niallia* sp. and *Bacillus* sp., respectively. Both strains were able to produce thermo- and alkali-stable mannanase enzymes. In our in vitro assay, Man26 and Man122 showed high catalytic activity on mannan-rich substrates such as locust bean gum and copra meal. The isolates could potentially be used as probiotics together with MOS as synbiotics to improve the utilization of MOS. The synergistic effects on shrimp growth performance and disease resistance are being further explored.

## Figures and Tables

**Table 1:** Morphological characteristics of Man26 and Man122 isolates.

Characteristics	Man26	Man122
**Cell morphology**	Rod shape, Gram stain positive 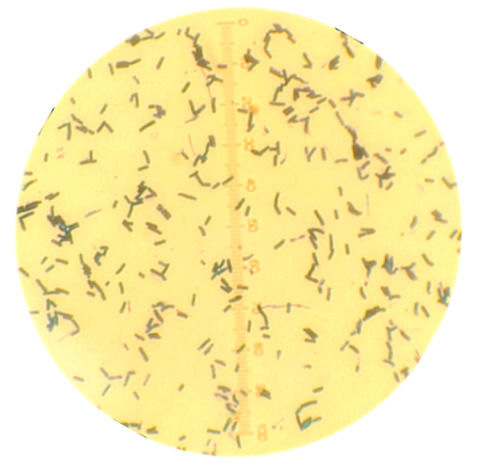	Short rod shape, Gram stain positive 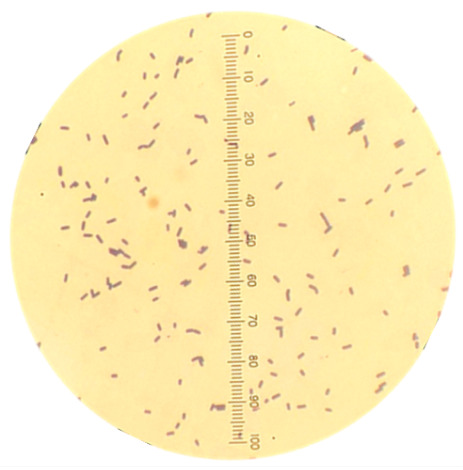
**Colony morphology**		
Colony color	White, shiny	White, dull
Surface	-	Mucoid
Margin	Regular	Irregular
Elevation	Swell	Flat
**Catalase**		
24 h	Positive	Positive
48 h	Positive	Positive

**Table 2:** Enzyme properties and kinetic parameters of crude mannanase from Man26 and Man122 for mannanase activity test using locust bean gum (LBG) as a substrate.

Source	Optimal pH	Optimal Temperature (^o^C)	*V_max_* (U·mg^−1^)	*K_m_* (mg·mL^−1^)
Man26	8.0	60	5.52 ± 0.70	18.92 ± 4.36
Man122	8.0	50	41.15 ± 12.33	34.53 ± 14.26

**Table 3:** Substrate specificity of crude mannanase produced from Man26 and Man122.

Substrates	Relative Activity (%)		*p* Value (*t*-test)
	Man26	Man122	
Locust bean gum (LBG)	100 ± 5.51 ^a^	100 ± 3.42 ^a^	-
Guar gum	32.97 ± 0.37 ^b, x^	19.13 ± 2.53 ^cd, y^	0.003
Carboxymethyl cellulose (CMC)	2.52 ± 1.45 ^e, x^	23.16 ± 1.19 ^c, y^	0.002
Avicel	18.31 ± 3.47 ^cd, x^	4.93 ± 3.66 ^f, y^	0.005
Xylan (larchwood)	1.95 ± 0.88 ^e, x^	15.78 ± 1.46 ^de, y^	0.008
Xylan (birchwood)	14.81 ± 1.57 ^d, x^	12.82 ± 1.53 ^e, x^	0.354
Xylan (oat spelts)	1.54 ± 1.23 ^e, x^	8.09 ± 0.6 ^f, y^	0.004
Potato starch	22.03 ± 1.95 ^c, x^	33.24 ± 1.74 ^b, x^	0.074

^a,b,c,d,e,f^ Superscripts indicate significant differences (*p* < 0.05) in each crude enzyme within the same columns. ^x,y^ Superscripts indicate significant differences (*p* < 0.05) between Man26 and Man122 using *t*-test. Mean ± standard deviations were calculated from triplicate samples (n = 3).

**Table 4:** Effects of chemicals on enzyme activities of crude mannanase produced from Man26 and Man122. The control was the enzyme reaction of each enzyme without chemicals.

Chemicals	Relative activity (%)		*p* Value (*t*-test)
	Man26	Man122	
Control	100 ^ab^	100^a^	-
10 mM CaCl_2_	67.36 ± 0.15 ^cd, x^	71.31 ± 1.01 ^d, y^	0.017
10 mM KCl	97.51 ± 0.18 ^ab, x^	91.34 ± 0.25 ^b, x^	0.219
10 mM MgCl_2_	105.38 ± 0.71 ^a, x^	67.81 ± 0.65 ^d, y^	0.016
10 mM MgSO_4_	93.37 ± 0.32 ^e, x^	60.35 ± 0.8 ^e, y^	0.022
10 mM NaCl	93.52 ± 0.49 ^b, x^	85.75 ± 0.34 ^c, x^	0.277
10 mM Na_2_SO_3_	63.41 ± 0.38 ^d, x^	26.05 ± 0.52 ^f, y^	0.014
10 mM NH_4_Cl	74.09 ± 0.15 ^c, x^	61.47 ± 0.61 ^e, y^	0.011
50 mM EDTA	43.54 ± 0.2 ^e, x^	4.53 ± 0.13 ^g, y^	0.014
1% SDS	24.55 ± 0.5 ^f, x^	1.38 ± 0.34 ^g, y^	0.015
5% SDS	23.66 ± 0.12 ^f, x^	0.36 ± 0.03 ^g, y^	0.002
10% SDS	20.56 ± 0.17 ^f, x^	0.31 ± 0.05 ^g, y^	0.003

^a,b,c,d,e,f,g^ Superscripts indicate significant differences (*p* < 0.05) in each crude enzyme within the same column. ^x,y^ Superscripts indicate significant differences (*p* < 0.05) between Man26 and Man122 using *t*-test. Mean ± standard deviations were calculated from triplicate samples (n = 3).

**Table 5:** Nutrient released from different feedstuffs under incubation with crude mannanase from Man26.

%	Substrates^Ψ^	Crude Mannanase of Man26					
		−	+	−	+	−	+
		Reducing sugar (Mg) ^Ψ^		Total Sugar (mg) ^Ψ^		Protein Release (mg) ^Ψ^	
1%	Palm kernel cake (PKC)	2.42 ± 0.22	20.15 ± 0.48 *	14.05 ± 1.24	28.53 ± 14.57	5.33 ± 0.12	8.55 ± 0.26 *
	Copra meal (CPM)	0.76 ± 0.18	28.71 ± 0.60 *	8.83 ± 0.84	33.21 ± 21.17 *	5.70 ± 0.61	8.93 ± 0.20 *
5%	Palm kernel cake (PKC)	13.84 ± 0.55	52.58 ± 1.62 *	61.74 ± 1.48	79.19 ± 24.94	7.13 ± 0.28	9.60 ± 0.45 *
	Copra meal (CPM)	4.12 ± 0.31	88.14 ± 2.48 *	35.98 ± 4.37	84.26 ± 54.21	5.61 ± 0.23	9.03 ± 0.13 *
1%	Maize	3.69 ± 0.30	15.54 ± 0.36 *	7.99 ± 1.54	24.29 ± 18.25	6.16 ± 0.24	9.15 ± 0.35 *
	Broken rice	17.53 ± 0.94	58.69 ± 2.30 *	21.56 ± 0.49	54.26 ± 25.39 *	4.77 ± 0.33	8.34 ± 0.52 *
	Soybean	0.18 ± 0.07	8.07 ± 1.04 *	46.85 ± 2.71	59.01 ± 11.49	9.29 ± 1.16	14.62 ± 0.26 *
	Feed	5.09 ± 1.19	5.95 ± 1.44	24.89 ± 6.49	25.74 ± 5.24	8.14 ± 0.07	10.75 ± 0.26 *
5%	Maize	61.88 ± 2.29	81.09 ± 1.40 *	69.55 ± 4.25	89.25 ± 15.28 *	7.41 ± 0.26	10.10 ± 0.10 *
	Broken rice	239.65 ± 7.81	261.29 ± 2.85 *	245.05 ± 26.36	259.64 ± 70.81	8.69 ± 0.19	10.72 ± 0.34 *
	Soybean	15.28 ± 4.37	21.81 ± 3.90 *	164.26 ± 4.26	190.63 ± 26.31	10.70 ± 0.15	15.49 ± 0.27 *
	Commercial shrimp feed	9.68 ± 1.01	11.05 ± 0.56	81.35 ± 1.67	84.20 ± 6.00	10.96 ± 0.30	13.43 ± 0.30 *

* The asterisk indicates a significant difference from each control counterpart (without mannanase) (*p* < 0.05) according to paired-samples *t*-test. The + and – signs indicate the reaction with and without crude mannanase, respectively. Mean ± standard deviations were calculated by triplicate (n = 3). ^Ψ^ Percent substrate content was calculated as the amount of substrate (g) per 100 mL of total reaction volume. The content of reducing sugar, total sugar, and protein release was expressed as mg per 50 mL of reaction volume

**Table 6:** Nutrient released from different feedstuffs under incubation with crude mannanase from Man122.

%	Substrates^Ψ^	Crude Mannanase of Man122					
		−	+	−	+	−	+
		Reducing Sugar (mg) ^Ψ^		Total Sugar (mg) ^Ψ^		Protein Release (mg) ^Ψ^	
1%	Palm kernel cake (PKC)	1.58 ± 0.25	12.30 ± 0.59 *	14.38 ± 0.74	24.17 ± 2.06 *	5.15 ± 0.24	10.31 ± 0.18 *
	Copra meal (CPM)	0.80 ± 0.73	30.47 ± 0.87 *	11.05 ± 2.52	49.19 ± 2.85 *	5.79 ± 0.31	8.91 ± 0.05 *
5%	Palm kernel cake (PKC)	15.29 ± 2.17	50.90 ± 3.50 *	54.14 ± 2.71	97.00 ± 7.25 *	7.32 ± 0.52	11.15 ± 0.23 *
	Copra meal (CPM)	5.49 ± 1.75	197.15 ± 5.49 *	31.71 ± 0.79	394.60 ± 23.77 *	6.08 ± 0.05	12.06 ± 0.44 *
1%	Maize	4.95 ± 0.34	17.07 ± 2.06 *	7.87 ± 2.43	36.58 ± 4.25 *	6.04 ± 0.22	9.36 ± 0.22 *
	Broken rice	27.08 ± 1.52	34.99 ± 4.46 *	31.02 ± 1.82	47.03 ± 3.73 *	4.85 ± 0.06	8.33 ± 0.16 *
	Soybean	0.23 ± 0.13	11.68 ± 0.50 *	47.45 ± 2.11	48.26 ± 2.40	8.31 ± 0.16	14.78 ± 0.20 *
	Feed	0.09 ± 0.05	22.56 ± 0.58 *	18.20 ± 1.62	50.00 ± 3.60 *	7.57 ± 0.26	11.2 ± 0.07 *
5%	Maize	67.68 ± 1.10	85.68 ± 26.14	77.72 ± 5.88	120.84 ± 32.93 *	7.25 ± 0.54	9.61 ± 0.07 *
	Broken rice	184.09 ± 11.75	322.85 ± 17.69 *	205.05 ± 18.95	382.94 ± 15.27 *	7.11 ± 0.44	11.07 ± 0.69 *
	Soybean	11.62 ± 4.05	33.56 ± 1.83 *	169.82 ± 12.65	178.56 ± 14.96 *	9.42 ± 1.05	15.36 ± 0.23 *
	Commercial shrimp feed	6.40 ± 2.98	33.46 ± 8.44 *	78.23 ± 2.62	137.90 ± 13.47 *	9.28 ± 0.43	13.13 ± 0.18 *

* The asterisk indicates a significant difference from each control counterpart (without mannanase) (*p* < 0.05) according to paired-samples *t*-test. The + and – signs indicate the reaction with and without crude mannanase, respectively. Mean±standard deviations were calculated by triplicate (n = 3). ^Ψ^ Percent substrate content was calculated as the amount of substrate (g) per 100 mL of total reaction volume. The content of reducing sugar, total sugar, and protein release was expressed as mg per 50 mL of reaction volume.

## Data Availability

Not applicable.

## Supplementary Materials

The following supporting information can be downloaded at: https://www.mdpi.com/article/10.3390/ani12192583/s1, Figure S1: Overview of experimental design to evaluate mannan oligosaccharides (MOS) from copra meal as shrimp feed supplements; Figure S2: Growth performance analysis of shrimp fed with commercial diet supplemented with 0%, 0.2%, 0.3% and 0.4% MOS. (A) final weight, (B) average daily growth, and (C) feed conversion ratio. The error bar represents a standard deviation value; Figure S3: Activity analysis of positive colonies stained with Congo red. Positive control was known mannanase producing bacteria. Negative control was non-mannanase producing bacteria, *Escherichia coli*.
