# Supplementary material for: Isolation and Characterization of Mannanase-Producing Bacteria for Potential Synbiotic Application in Shrimp Farming
Source: Animals (Basel). 2022 Sep 27;12(19):2583. doi: 10.3390/ani12192583 (PMC9558954; doi:10.3390/ani12192583)

Figure S1. Overview of experimental design to evaluate mannan oligosaccharides (MOS) from copra meal as shrimp feed supplements.

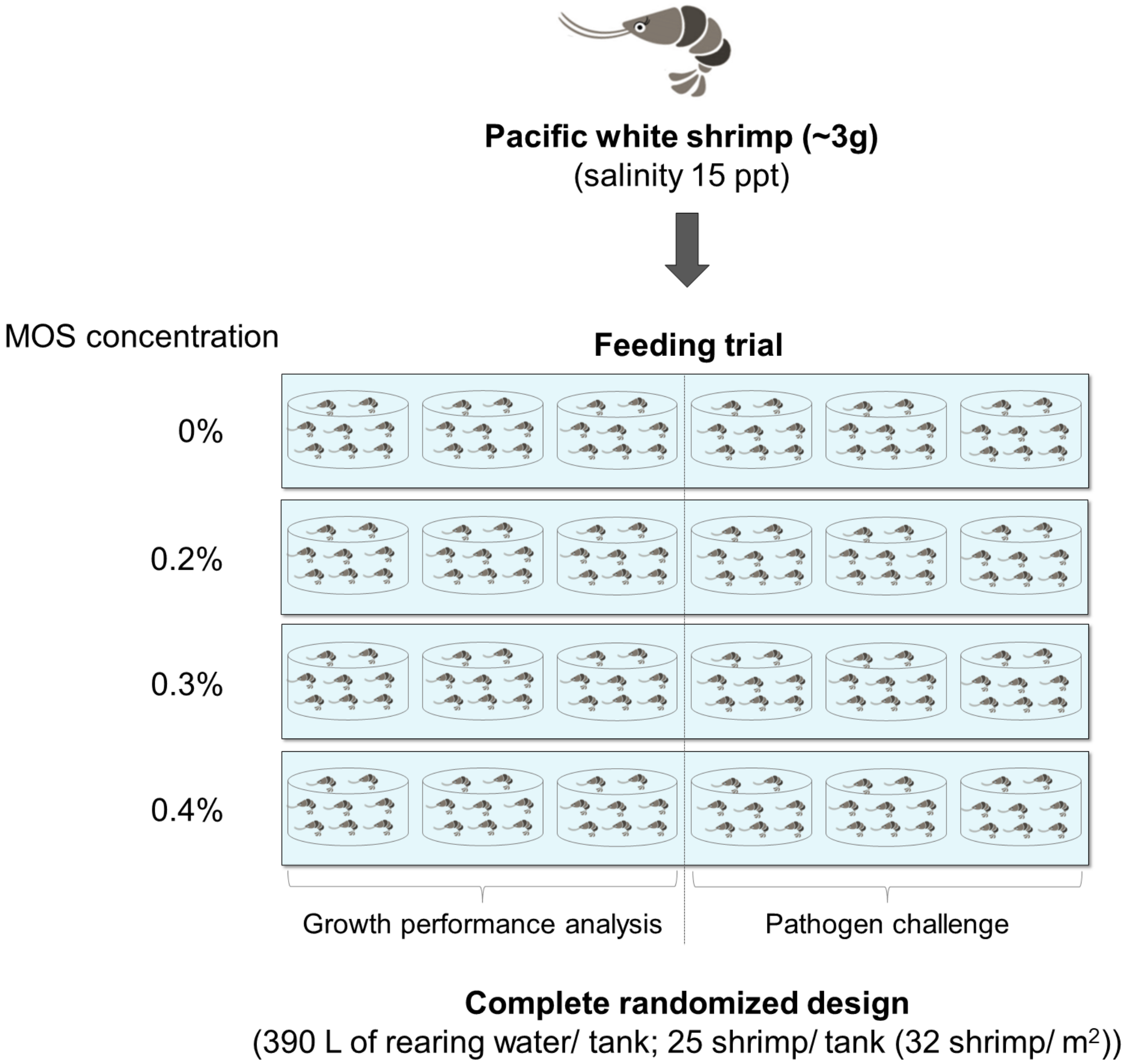

Figure S2. Growth performance analysis of shrimp fed with commercial diet supplemented with 0%, 0.2%, 0.3% and 0.4% MOS. (A) final weight, (B) average daily growth, and (C) feed conversion ratio. The error bar represents a standard deviation value.

A

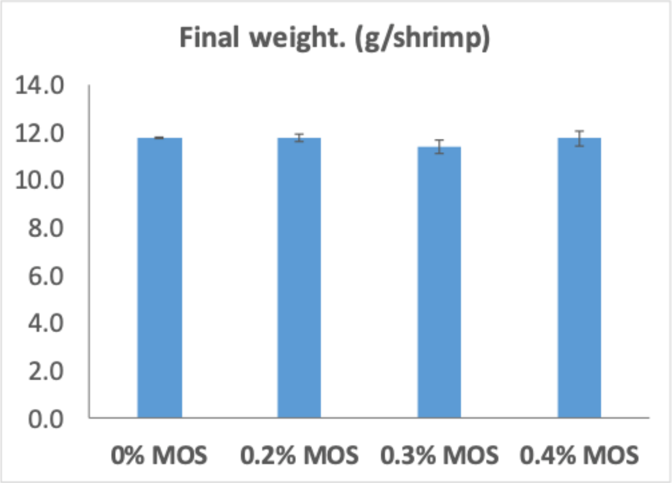

B

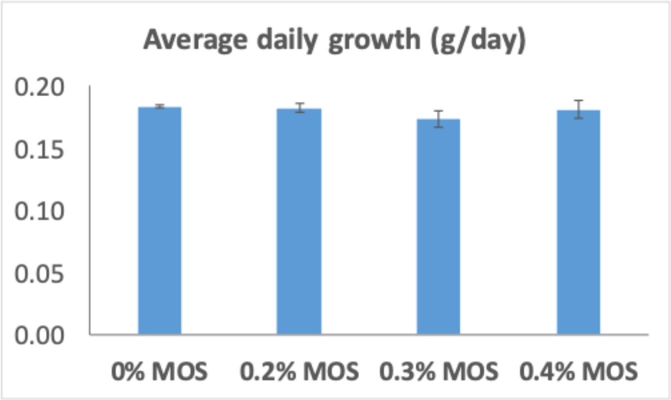

C

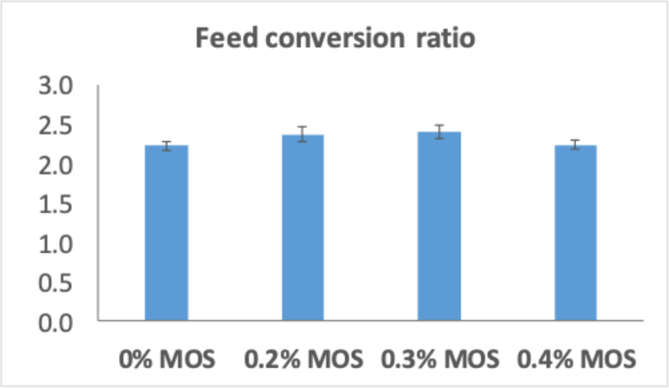

Figure S3. Activity analysis of positive colonies stained with Congo red. Positive control was known mannanase producing bacteria. Negative control was non-mannanase producing bacteria, *Escherichia coli*.

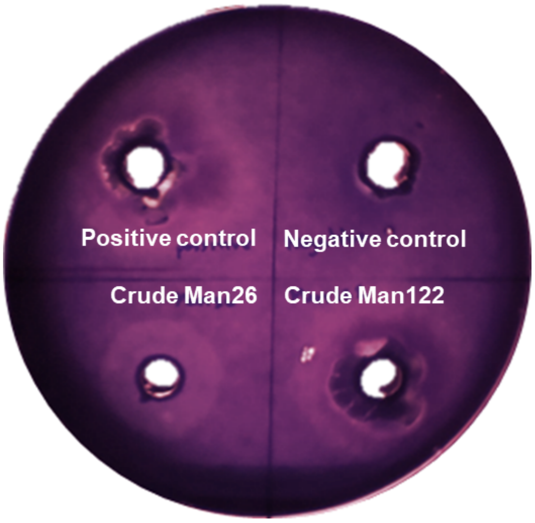

Supplement: Supplementary file 1 [file animals-12-02583-s001.zip › animals-1868560-Supplementary.pdf]
